# Effects of reservoir mechanical properties on induced seismicity during subsurface hydrogen storage

**DOI:** 10.1098/rsta.2023.0187

**Published:** 2024-08-09

**Authors:** J. E. J. Burtonshaw, A. Paluszny, A. Mohammadpour, R. W. Zimmerman

**Affiliations:** ^1^ Department of Earth Science and Engineering, Imperial College London, London, UK

**Keywords:** hydrogen, storage, seismicity, slip

## Abstract

The intermittent storage of hydrogen in subsurface porous media such as depleted gas fields could be pivotal to a successful energy transition. Numerical simulations investigate the intermittent storage of hydrogen in a porous, depleted subsurface reservoir. Various parametric studies are performed to assess the effect of mechanical properties of the reservoir (i.e. Young’s modulus, Poisson’s ratio, Biot coefficient and permeability) on the induced fault slip of a single through-going fault that transverses the entire reservoir. Simulations are run using a three-dimensional, finite element, fully coupled hydromechanical code with explicit representations of layers and faults. The effect of the domain mesh refinement and fault mesh refinement on the fault slip versus operation time solution is investigated. The fault is observed to slip in two distinct events, one during the second injection period and one in the third injection period. The fault is not observed to slip during the storage or withdrawal periods. It is found that in order to minimize seismic risk, a reservoir rock with high Young’s modulus (>40 GPa), high Poisson’s ratio (>0.30) and high Biot coefficient (>0.65) would be preferable for hydrogen storage. Reservoir rocks of low Young’s modulus (10–30 GPa), intermediate Poisson’s ratio (0.00–0.30) and low-to-intermediate Biot coefficient (0.25–0.65), at high injection rates, were found to have higher potential of inducing large seismic events.

This article is part of the theme issue ‘Induced seismicity in coupled subsurface systems’.

## 1. Introduction

Hydrogen as an energy carrier is attracting growing interest from governments, major energy companies and researchers owing to its very high energy density, versatility in production routes, compatibility with renewable energy systems and potential for large-scale energy storage.

The European Commission of the European Union reports that up to 25% of future renewable electricity will go towards the production of green hydrogen by 2050 [1]. The International Renewable Energy Agency (IRENA) and the Hydrogen Council (HC) estimate that hydrogen will constitute 6 [2] and 18% [3] of the global energy mix by 2050, respectively [4]. The United States Energy Information Administration (EIA) reports that global annual energy consumption will increase by nearly 50% from 2018 levels to approximately 267 000 TWh by 2050 [5]. From the hydrogen energy share estimates of IRENA and HC and the global energy consumption estimates of the EIA, this translates to 16 000–48 000 TWh per year of energy from hydrogen being required by 2050. It follows that hydrogen will need to be stored on an enormous scale (ideally multiple TWhs in any given project).

Currently, no existing method of hydrogen storage can realistically satisfy such demand. Methods of hydrogen storage can be categorized into one of the following two groups: physical storage or material/chemical storage. Physical storage includes (i) compressed hydrogen gas (CGH_2_) in tanks at pressures typically between 35 and 70 MPa and ambient temperature [6], (ii) cooled liquid hydrogen (LH_2_) in tanks at temperatures lower than −253°C and at ambient pressure [7], (iii) cryo-compressed liquid hydrogen (CcH_2_) in tanks at temperatures lower than −253°C and at typical pressures of 35 MPa [8], and (iv) injection of gaseous hydrogen into subsurface geological media, either engineered or natural salt caverns, engineered steel-lined rock caverns or natural porous media such as aquifers, depleted gas fields and depleted oil fields (table 1). Material and chemical storages include (i) adsorbents (i.e. MOF-5, carbon nanotubes and buckminsterfullerene [20,21]) where hydrogen is held to the surface of the compound by physisorption; (ii) liquid organics (i.e. BN-methylcyclopentane [22]) where the compound is dehydrogenated usually under intermediate temperatures (i.e. 80°C) and under the action of a catalyst (i.e. FeCl_2_); (iii) interstitial hydrides (i.e. La-Ni hydride [23,24]) where chemisorption of hydrogen to metal (i.e. Ni in La-Ni hydride) atoms causes H_2_ dissociation, and the produced H^+^ ions diffuse into the alloy-hydride lattice; (iv) complex hydrides (i.e. sodium alanate [25,26]) which are metal salts where the anion contains a hydride, and which can be dehydrogenated for hydrogen production typically at high temperatures (i.e. 200°C) under the action of a titanium catalyst; and (v) chemical hydrogen (i.e. ammonia borane [27]) which are hydrogen-containing compounds that are capable of undergoing hydrolysis.

**Table 1.** All known current and past geological hydrogen storage projects.

location	storage type	gas composition (%)	pressure (MPa)	temperature (°C)	volume (m ${\;}^{3}$ )	depth (m)	status
Beynes, FR	aquifer	50% H_2_ [9]	—	—	—	430 [9]	1956/1972 [9]
Clemens Dome, US	salt cavern	95% H_2_ [9]	7−13.5 [10]	—	580 × 10^3^ [11]	1000–1300 [11]	1983 [10]
Diadema/Hychico, AR	depleted gas field	10% H_2_ [9]	1 [9]	50 [9]	600 × 10^3^ [12]	600 [9]	2009—unk [9]
Ketzin, DE	aquifer	62% H_2_ and CO_2_ [9]	7.7–7.8 [13]	—	—	200–250 [9]	2008/2010 [13]
Kiel, DE	salt cavern	62% H_2_ [9]	8−10 [9]	—	32 × 10^3^ [9]	1330 [14]	1971 [14]
Lobodice, CZ	aquifer	50% H_2_ and 25% CH_4_ [9]	9 [9]	34 [9]	—	430 [9]	1965 [9]
Moss Bluff, US	salt cavern	—	5.5–15.2 [10]	—	566 × 10^3^ [11]	335–1400 [11]	2007 [10]
Spindletop, US	salt cavern	95% H_2_ [9]	Conf. [15]	—	—	1500 [16]	2014 [15]
Teesside, UK	salt cavern	95% H_2_ and 3–4% CO_2_ [9]	4.5 [10]	—	70 × 10^3^ (3 × ) [11]	370 [11]	1972 [10]
Underg. Sun Storage, AT	depleted gas field	10% H_2_ [9]	7.8 [9]	40 [9]	1200 × 10^3^ [17]	1000 [9]	2015/2017 [9,18]
Yakshunovskoe, RU	salt cavern	—	—	—	—	—	unk (2010) [19]

The four minor salt cavern hydrogen storages in Germany (Engelbostel, Bad Lauchstädt, Hähnlein and Eschenfelden) are excluded from this table.

Experience of geological storage of hydrogen in subsurface rock systems is very limited, and geological storage of pure hydrogen in porous media is yet to be implemented. To date, six known storage projects involving H_2_ gas have been implemented in natural or engineered salt caverns (table 1). A salt cavern is an empty void created via designed dissolution or naturally existing within salt deposits. Salt caverns have stored gas of the highest hydrogen composition (relative to aquifers and oil/gas fields), but the volumes stored are typically low (<600 000 m^3^), the depths of storage are reasonably shallow (<1500 m) and the cavern pressures are low (<15 MPa). There are only three major examples of hydrogen storage in aquifers (table 1), with four further minor examples in Germany [28]. In all cases, the hydrogen composition is typically close to 50% and is thus much lower in purity than the examples in salt caverns. The depths of storage are very shallow (<500 m), and the pressures (<10 MPa) and temperatures (<50°C) of storage are also low. In all cases, except that of Ketzin, the storage site was opened to store town gas, a product of coal gasification, existing as a mixture of gases (CH_4_, H_2_, CO_2_, CO and others) and the precursor to natural gas in heating and energy systems. Finally, there are only two case studies of gas storage involving hydrogen within depleted hydrocarbon fields (table 1). Both have occurred in depleted gas fields; no hydrogen has ever been stored in a depleted oil field. The stored gas in both cases consisted of only 10% hydrogen, and as such, little can be learned from such storage projects for future geological hydrogen storage in depleted hydrocarbon fields. In the case of Diadema, the hydrogen was not intended for storage; it was merely injected to be bio-converted into methane for later production.

Despite there being less experience of hydrogen storage geologically, relative primarily to other physical-storage techniques (i.e. CGH_2_, LH_2_ and CcH_2_), but also to some material/chemical storages, hydrogen storage in depleted oil and gas reservoirs is the most promising method of both efficiently storing hydrogen and storing enough quantity to satisfy the 16 000–48 000 TWh of future (2050) demand. Consider a depleted hydrocarbon reservoir, with size and characteristics as follows: porosity, ϕ = 0.15; thickness, h = 150 m; area, A = 2000 × 2000 m^2^; hydrogen saturation, $S_{h}$ = 0.75; hydrogen recovery factor, $R_{f}$ = 0.8; hydrogen density, $\rho_{h}$ = 9 kg/m^3^. The mass of recoverable stored hydrogen is then $\rho_{h}Ah\phi S_{h}R_{f}$ = 486 000 000 kg. A total of 1 kg of hydrogen equates to 33.3 kWh of energy [29]. Thus, one typical depleted hydrocarbon field can store approximately 16.2 TWh of energy or 1% of the total annual energy consumption of the United Kingdom. This poses the question: How many of the other storage methods are required to equate to such quantities of energy storage? One of MaHyTec’s largest CGH_2_ tanks (dimensions: 0.84 × 1.87 m) can store 4.2 kg of hydrogen. Therefore, over 115 million of such tanks would be required to match the energy storage capacity of a single depleted hydrocarbon reservoir. A total of 115 million of these tanks would occupy almost the entire land surface of the city of Glasgow. Following the stoichiometry of Scheme 1 given in Luo *et al*. [22], 31 million tonnes of liquid organic (BN-cyclomethylpentane) would be required to match the energy storage capacity of a single depleted oil/gas field. In contrast, that is not dissimilar to the amount of natural gas (assuming CH_4_ composition) consumed by the entire United Kingdom in 2017 (52 million tonnes). Finally, salt caverns have not stored more than 600 000 m^3^ (table 1). A salt cavern of 600 000 m^3^ at a typical pressure of 10 MPa would store hydrogen with an approximate density of 7 kg/m^3^ [30]. This corresponds to one salt cavern having a storage capacity of 4 200 000kg. Therefore, to store the same quantity of hydrogen as a single typical depleted hydrocarbon field, 116 typical salt caverns would be required. Thus, it is clear that geological hydrogen storage in porous media is the most volumetrically efficient way of storing hydrogen and the only realistic technique of storing sufficient quantities to meet future demand.

Future research problems in geomechanics have been identified by numerous authors (i.e. [30]), but to date, no studies of key geomechanical phenomena resulting from hydrogen storage have been performed. Such problems include induced seismicity, mechanical integrity of the caprock (which differs in H_2_ storage relative to CO_2_ storage owing to the fatigue induced from the cyclical injection and withdrawal of the fluid) and surface subsidence. In particular, induced seismicity from subsurface injection has become a topic of public and regulatory interest. For example, public pressure in the United Kingdom owing to very small magnitude (*M*_*L*_ 1.6 and 2.9) seismic events at the Preston New Road site in Lancashire [31] has resulted in a nationwide ban on hydraulic fracturing, despite the promise of energy independence that it offers. For geological hydrogen storage in depleted fields, especially onshore, to be operationally sustainable far into the future, the potential for seismic events must be fully assessed and qualified prior to implementation.

This article performs an analysis of geomechanical issues associated with hydrogen storage in depleted hydrocarbon fields by assessing the potential for induced seismicity during the storage of gaseous hydrogen. In particular, the effects of mechanical properties, including Young’s modulus, Poisson’s ratio, Biot coefficient and matrix permeability, on seismic event potential within a depleted gas field are numerically explored. Induced seismicity is quantified in terms of tangential fault slip and potential moment magnitude. The modelled depleted gas field is designed to be analogous to typical North Sea depleted hydrocarbon reservoirs.

## 2. Effects of reservoir properties on fault slip

Fluid mechanical, operational and rock mechanical properties can all influence fault slip and induced seismicity during both the injection and withdrawal of a fluid. This section reviews previous studies from geo-energy fields examining the effects of varying mechanical properties on inferred fault slip, in particular Young’s modulus, Poisson’s ratio, Biot coefficient and matrix intrinsic permeability. A summary of some of the findings in this section is provided in table 2, which also summarizes findings of the effects of varying fluid mechanical (i.e. fluid viscosity, fluid compressibility and fluid density), operational (i.e. injection rate and withdrawal rate), fault (i.e. dip and length) and other mechanical (i.e. rock compressibility and *in situ* stresses) properties on induced fault slip and seismicity.

**Table 2.** Summary of the literature review.

property	numerical method	geo-energy type	method	cases	fault loc.	mode	effect on slip
**mechanical**							
Young’s modulus	2D coupled HM FEM [32] and 3D coupled HM FEM [33]	hydraulic fracturing [32,33]	fault slip [32] and CFS [33]	5–50 GPa [32] and 2–7 GPa [33]	res [32,33]	inj [32,33]	decreases [32,33]
Poisson’s ratio	2D coupled HM FEM [32,34]	natural gas production [34] and hydraulic fracturing [32]	Mohr–Coulomb [34] and fault slip [32]	0.10–0.25 [34] and 0.10–0.30 [32]	res [32,34]	prod [34] and inj [32]	decreases [34] and insensitive [32]
rock compressibility	2D coupled HM FEM [35]	fluid injection [35]	qualitative seismicity type [35]	10^−11^–10^−10^ Pa^−1^ [35]	res [35]	inj [35]	insensitive [35]
Biot coefficient	3D coupled HM FEM [33,36]	wastewater [36] and hydraulic fracturing [33]	CFS [33,36]	0.44 and 0.79 [36], 0.22–0.66 [33]	base [36] and res [33]	inj [33,36]	decreases slightly [33,36]
permeability [37]	2D coupled THM FEM [37]	CO_2_ storage [37]	fault slip [37]	10^−12^–10^−14^ m^3^/s [37]	res [37]	inj [37]	time to failure decreases [37]
porosity	2D coupled THM FEM [37]	CO_2_ storage [37]	fault slip [37]	0.10–0.20 [37]	res [37]	inj [37]	insensitive [37]
**hydraulic**							
fluid compressibility	2D coupled HM FEM [35]	fluid inj [35]	qualitative seismicity type [35]	10^−11^–10^−7^ Pa^−1^ [35]	res [35]	inj [35]	decreases [35]
inj rate	2D spring-slider [38] and Cyl granite samples [39]	fluid injection [38,39]	critical stiffness analysis [38] and strain gauge/AE hypocentre analysis [39]	0.25–1.0 [38] and 0.2–0.8 ml/min [39]	res [38,39]	inj [38,39]	increases [38 ,39]
production rate	2D coupled HM FEM [34] and Bayesian change point [40,41]	natural gas production [34,40,41]	fault slip [34] and statistical analysis [40,41]	2.5–10 m^3^/s [34]	res [34]	prod [34,40,41]	insensitive [34] and increases [40,41]
**fault**							
friction coefficient	2D coupled HM FEM [42, 43]	hydraulic fracturing [42] and water inj [40]	fault slip [42] and poroelastic stress change [43]	0.20–0.60 [42] and 0.10–0.75 [43]	res [42] and base [43]	inj [42,43]	insensitive [42] and decreases [43]
fault permeability	2D coupled THM FEM [44] and 3D coupled THM FEM [45]	CO_2_ storage [44] and geothermal [45]	fault slip [44,45]	10^−15^–10^−17^ m^3^/s [44] and 10^–12^–10^−16^ m^3^/s [45]	res [44,45]	inj [44,45]	insensitive—time to failure increases [44] and generally decreases [45]
fault length	2D coupled HM FEM [32]	hydraulic fracturing [32]	fault slip [32]	10–100 m [32]	res [32]	inj [32]	increases [32]
**stress**							
$\sigma_{H}/\sigma_{V}$	2D coupled THM FEM [44]	CO_2_ storage [44] and hydraulic fracturing [46]	fault slip [44,46]	0.65–0.70 [44] and 0.50–0.55 [46]	res [44,46]	inj [44,46]	decreases [44,46]

The ‘effect on slip/seismicity’ column refers to the subsequent effect on slip/seismicity as that parameter increases in value.

### (a) Poisson’s ratio

Zbinden *et al*. [34] demonstrated that for the same production rate, for a given value of Young’s modulus, as Poisson’s ratio was reduced, the likeliness of intersecting the Mohr–Coulomb failure line increased owing to the fact that at smaller Poisson’s ratios, the differential stress is becoming larger. Therefore, Poisson’s ratio is likely to be an important parameter in controlling production-induced earthquakes. Meng *et al*. [32] implemented a two-dimensional 500 × 500 m plane-strain hydraulic fracturing model containing a 50 m length natural fracture at 45° to the water injection wellbore. They varied the Poisson’s ratio between 0.1 and 0.3 and found that as the Poisson’s ratio decreases, the fracture slip increases for a given value of the Young’s modulus. However, the difference in slip was on the sub-centimetre scale and, as such, is likely below the numerical precision of the simulator. At high Young’s moduli of greater than 30 GPa, the difference in slip was a maximum of only 2 mm between Poisson’s ratios of 0.1 and 0.3. The difference in slip between the Poisson’s ratio cases increased as Young’s modulus decreased, with a maximal difference in slip of approximately only 12 mm between Poisson’s ratios of 0.1 and 0.3 at a Young’s modulus of 5 GPa. Essentially, the fault slip was insensitive to the tested range of Poisson’s ratio. Therefore, it is implied that during fluid injection, Poisson’s ratio may not have a significant effect on fault slip.

### (b) Young’s modulus

Meng *et al*. [32] varied the Young’s modulus of the rock during water injection for hydraulic fracturing and assessed the slip along the fault in their model, where the borehole intersects. They found a very strong dependence of slip on Young’s modulus, which varied between 5 and 50 GPa. The slip was shown to decrease as Young’s modulus increased, with approximately 120 mm difference in the slip between Young’s moduli cases of 5 and 50 GPa. For Young’s moduli > 30 GPa, the difference in slip is negligible ( < 7.5 mm), but Young’s modulus appears to be an important parameter affecting fault slip when it takes a value of < 30 GPa. Hui *et al*. [33] performed a sensitivity analysis on fault slip under varying mechanical and operational properties within a 4000 × 4500 × 500 m four-layer hydraulic fracturing model containing a single fault that is penetrated by two horizontal wellbores. The four layers were constructed to be representative of the Duvernay, Swan Hills, Cambrian and Precambrian formations within the Duvernay East Shale Basin that had previously experienced a local magnitude 4.18 induced earthquake from hydraulic fracturing. They did not quantify the slip but instead measured the change in the Coulomb failure stress (CFS) between the cases. They varied Young’s modulus between 2.17 and 6.52 GPa and found that as Young’s modulus increased, the change in CFS decreased, implying that the likelihood of seismicity decreases as Young’s modulus increases, which is consistent with results reported by Meng *et al*. [32]. However, the difference in the change in CFS between the cases was small at only 0.5 MPa between the 2.17 and 6.52 GPa cases. The sensitivity analysis ranked Young’s modulus as a much less important factor on fault slip than the distance between the fault and hydraulic fractures, the permeability of the fault and the injection rate, but slightly more important than the reservoir permeability and the Biot coefficient.

### (c) Biot coefficient

Fan *et al*. [36] conducted three-dimensional finite element simulations in ABAQUS to assess seismic fault reactivation along a 20 m width fault within a crystalline basement unit when injecting wastewater into an overlying sedimentary reservoir. They varied the Biot coefficient in two separate cases, α = 0.79 and α = 0.44, and assessed the impact on the CFS. They found that a decrease in the reservoir Biot coefficient caused a decrease in induced normal stress and thus an increase in induced CFS, meaning that as reservoir Biot coefficient decreases, the probability of slip increases. In the hydraulic fracturing work of Hui *et al*. [33], in the model resembling the Duvernay East Shale Basin, they varied the Biot coefficient between 0.22 and 0.66 and also found that as Biot coefficient decreases, the change in CFS increases slightly, thus concluding again that as Biot coefficient decreases, the probability of slip increases. They also concluded that in a sensitivity analysis of the fault-hydraulic fracture separation that included the fault permeability, the injection rate, Young’s modulus, the Biot coefficient and the reservoir permeability, the Biot coefficient had the least impact on CFS and thus the least impact on slip of that parameter list. However, poroelastic stress perturbations have been recognized as a mechanism by which seismicity can be induced [43,47]. Changes in pore pressure generate poroelastic stresses, which alter the total stress state of the reservoir and the surrounding lithological units, potentially inducing slip. While pore pressure diffusion resulting in effective normal stress reduction is the dominant mechanism for inducing slip in conductive faults within the reservoir or in conductive faults that are hydraulically connected to the reservoir, poroelastic stresses dominate slip on low-permeability faults within the basement unit. As such, poroelasticity can be a key mechanism for driving slip at further distances from the injection point beyond the region of elevated pore pressure. As such, it is remarkable that Hui *et al*. [33] deemed the Biot coefficient a largely insignificant parameter for governing the change in CFS and thus the likeliness of slip. No study appears to have directly assessed the impact of Biot coefficient on the fault slip, and it is important that any numerical method used to investigate induced seismicity has poroelastic coupling in which the pore pressure changes induce changes in stress and changes in stress induce changes in pore pressure.

### (d) Intrinsic permeability and porosity

Mortezaei & Vahedifard [37] conducted numerical two-dimensional single-phase CO_2_ injection experiments in a domain containing a 1 km length fault, in which the permeability of the reservoir unit was varied between 10^−12^ and 10^−14^ m^2^. They found that the fault slip was largest in the low-permeability case, owing to greater pore pressure build-up, but also found that the time to rupture was larger. Mortezaei & Vahedifard [37] also varied the reservoir porosity between 0.10 and 0.20 and found that slip was identical regardless of porosity.

## 3. Hydrogen fluid properties

Hydrogen has hydraulic properties that differ significantly from those of other fluids and injectants traditionally deployed for subsurface fluid injection. This section outlines and discusses the properties of gaseous hydrogen at a reservoir pressure of 15 MPa and a reservoir temperature of 90°C, which are the reservoir pressures and temperatures employed for the modelling in this work.

### (a) Density

The density of hydrogen at a pressure of 15 MPa and a temperature of 90°C is approximately 9 kg/m^3^. Within a typical range of reservoir pressures, 0–50 MPa, and a typical range of reservoir temperatures, 40–120°C, the density of hydrogen may range anywhere between 0 and 30 kg/m^3^ [30]. These are extremely low fluid densities relative to other geo-energy injectants over the same 0–50 MPa and 40–120°C pressure and temperature ranges, such as brine (1070–1160 kg/m^3^), carbon dioxide (190–850 kg/m^3^) and natural gas of CH_4_ composition (50–210 kg/m^3^). This article examines the influence of such low hydrogen densities on induced seismicity in the slightly larger range of 0–50 kg/m^3^ to also account for abnormally cold (<40°C) or abnormally high pressured (>50 MPa) reservoirs, as hydrogen density trends upward with lower temperature and higher pressure [30].

### (b) Viscosity

The viscosity of hydrogen is assumed to have values of between 9 and 12 µPa s for typical reservoir temperatures of 40–120°C and reservoir pressures of 10–50 MPa [30]. Hydrogen is much less viscous over the same temperature and pressure ranges than other fluids used in geo-energy applications such as carbon dioxide (20–75 µPa s) [30], methane-based natural gas (15–27 µPa s) [30], brine (400–1200 µPa s) [48] and hydraulic fracturing fluid (2000–1 000 000 µPa s) [49]. Hydrogen has a viscosity of 10.4 µPa s at a pressure of 15 MPa and temperature of 90°.

### (c) Compressibility

At 15 MPa and 60°C, the *Z*-factor for hydrogen is 1.06 [50]. The same study shows that the *Z*-factor, the measure of how much a real gas deviates from ideal behaviour, decreases with increasing temperature at 15 MPa. Thus, extrapolating implies a *Z*-factor of approximately 1.04 at 15 MPa and 90°C. In this case, since the *Z*-factor is very close to 1, we will assume it behaves in a sufficiently ideal way, so we can take the isothermal bulk modulus as the pressure. This would give an isothermal compressibility of 6.67 × 10^−8^ Pa^−1^. However, at higher reservoir pressures, this approximation is not reasonable. This leaves much uncertainty around the compressibility of hydrogen in its geological storage, and further experimental work may need to be done in this regard if compressibility is shown to be a key factor for geomechanical storage hazards.

## 4. Methodology

Simulations associated with this work are performed using a three-dimensional, fully coupled hydromechanical simulator based on linear elastic fracture mechanics theory and known as the Imperial College Geomechanics Toolkit (ICGT) [51,52]. The ICGT is a finite element method computational geomechanics framework, developed in C++, that has been developed and validated in the context of mechanical fracture contact and friction [53], fracture interaction [54,55], growth of fracture networks [52], hydraulic fracturing [56], advective–diffusive heat transfer in fractured geothermal media [57], heat conduction and transfer between injected fluid and rock in low-permeability oil and gas reservoirs [58] and hydrodynamics of CO_2_ injection-induced changes in fault geometry [59]. The code models deformation, fracture growth and flow through fractured media in three dimensions [60,61].

Matrix and fault flow are coupled through the inclusion of a mass transfer term owing to leak-off from the matrix to the fault. Friction within the fault surface is numerically resolved using the augmented Lagrangian method. Fluid flow is modelled within the matrix and the fault using a three-variable approach that captures displacement, matrix flow and fracture flow. Flow through the matrix and rock mechanical deformation are coupled via the concept of effective stress. Finally, rock mechanical deformation and fault flow are coupled by hydraulically loading fracture walls and ensuring the compatibility of fracture volumetric strains.

### (a) Stress and strain

Stress and strain are computed using the finite element method, according to


(4.1) $$\sigma =D(\varepsilon -\varepsilon_{0})+\sigma_{0}$$

where σ is the Cauchy stress tensor, D is the linear elastic stiffness matrix, ε is the infinitesimal strain tensor, $\varepsilon_{0}$ is the initial strain and $\sigma_{0}$ is the initial stress [62].

Since the system is in static equilibrium, Cauchy’s first law of motion


(4.2) ∇⋅σ=−F

must be obeyed, where ∇ is the divergence operator and F is a vector of the body forces acting on the system externally, per unit volume.

The Intel Pardiso direct solver is used to perform matrix inversion of the accumulated FEM matrix. The rock matrix is assumed to be homogeneous, isotropic and a linear-elastic medium.

### (b) Fault flow, matrix flow and deformation

Fluid flow through the bulk rock mass is modelled by combining mass conservation of the flowing fluid with Darcy’s law while neglecting inertial effects. The governing equation is as follows:


(4.3) $$div\left(\frac{k_{m}}{\mu_{f}}\left(\nabla P_{m}+\rho_{f}g\right)\right)=\left[\alpha \frac{\partial \left(divu\right)}{\partial t}+\left(\phi c_{f}+\frac{\alpha -\phi }{K_{s}}\right)\frac{\partial P_{m}}{\partial t}\right]+\frac{k_{n}}{\mu_{f}}\frac{\partial p_{m}}{\partial {\text{n}}_{c}}\delta \left(x-x_{c}\right).$$

The function $\delta \left(x-x_{a}\right)$ is the Dirac delta function, with $x_{a}$ specifying the position of the fracture, $c_{f}$ is the fluid compressibility, g is the acceleration owing to gravity, $k_{m}$ is the matrix permeability, $K_{s}$ is the matrix bulk modulus, $n_{c}$ is the unit normal pointing outward from the fracture wall, $p_{f}$ is the fluid pressure within the fracture, $p_{m}$ is the fluid pressure within the matrix, t is elapsed time, u is the displacement in the rock matrix, α is the Biot coefficient, Γ is the domain boundary, $\Gamma_{c}$ is the fracture boundary, Ω is the domain of interest, ϕ is the matrix porosity and $\rho_{f}$ is the fluid density.

Fluid flow through the fault assumes a laminar flow model constructed from lubrication theory. The governing equation is as follows:


(4.4) $$\nabla \cdot \left(\frac{a_{f}^{3}}{12\mu }\nabla p_{f}\right)=a_{f}c_{f}\frac{\partial p_{f}}{\partial t}+\frac{\partial a_{f}}{\partial t}-\frac{k_{m}}{\mu }\frac{\partial p_{m}}{\partial n_{c}},$$

where $a_{f}$ is the fault aperture and μ is the fluid viscosity.

Fluid flow in the fault, fluid flow in the bulk rock mass and poroelastic deformation of the rock mass are concomitantly accounted for by the ICGT. Equation (4.2) is transformed into equation (4.5), which governs the entire deformation field for a saturated rock mass, by combining effective stress and imposing boundary traction of hydraulic loading on fault walls, and subsequently integrating over each finite element. The final governing differential equation is thus


(4.5) $$\int_{\;\;\;\;\Omega }\left[\nabla \cdot \left(D\varepsilon -\alpha p_{m}I\right)+F\right]d\Omega =\int_{\;\;\;\;\Gamma_{c}}p_{f}n_{c}d\Gamma ,$$

where I is the identity matrix of second order [56].

### (c) Friction, tangential fault slip and contact

The fault is represented as a discrete discontinuity in the rock mass, bound by two smooth surfaces. While open, the fault is cohesion and traction-free. However, once the two fault surfaces contact one another, or intersect, a frictional contact constitutive law, derived from the Amontons–Coulomb first law of friction, governs the boundary conditions imposed on the fault surfaces. Gaps are examined between nodes on each side of the fault, and calculations of traction are performed over elements. The two fault surfaces are defined using a strict leader–follower formulation.

A normal gap function, $g_{N}$ , can be used to detect contact between the fault surfaces and enforce displacement constraints. The normal gap function measures the perpendicular gap between the two fault surfaces and is defined as


(4.6) $$g_{N}=\left(x^{f}-x^{l}\right)\cdot n=\left(u^{f}-u^{l}\right)\cdot n-{\hat{g}}_{N},$$

where x are position vectors of fault surfaces, u are displacement vectors of fault surfaces, n is the unit normal vector, ${\hat{g}}_{N}$ is a scalar defining the initial normal gap between the crack surfaces prior to any displacement and f and l are superscripts denoting ‘follower’ and ‘leader’ surfaces, respectively.

At the exact point in time when a node on the follower surface first touches the leader surface, $g_{N}$ is 0. Therefore, from equation (4.6), it can be written that ${\hat{g}}_{N}$ = ${\left(u^{f}-u^{l}\right)}_{c}\cdot n$ , where *c* is a subscript denoting ‘first contact’. Once first contact has occurred, an initial tangential gap vector, ${\hat{g}}_{T}$ , can be defined. It measures the tangential displacement between two equivalent points on both surfaces (one on the master leader surface and one on the follower slave surface) up to the point of contact and is calculated as ${\hat{g}}_{T}$ = $\left(I-n⊗n\right){\left(u^{f}-u^{l}\right)}_{c}$ , where I is the identity matrix of the third order. The tangential gap function, $g_{T}$ , which measures the displacement between equivalent points on the leader and follower surfaces in the direction of the leader surface after initial contact of the leader and follower fault surfaces, also referred to as fault slip, is given by


(4.7) $$g_{T}=\left(I-n⊗n\right)\left(x^{f}-x^{l}\right)=\left(I-n⊗n\right)\left(u^{f}-u^{l}\right)-{\hat{g}}_{T}.$$

The contact traction acting on the leader surface, $t_{l}$ , can be decomposed into a normal, p , and tangential, τ , traction: p = $t_{l}\cdot n$ and τ = $\left(I-n⊗n\right)t_{l}$ , respectively. The corresponding contact traction acting on the follower surface, $t_{f}$ , obeys Cauchy’s first law and thus must equal $-t_{l}$ . The tangential friction, τ , is defined according to one of two frictional states, based on the Amontons–Coulomb first law of friction


(4.8) $$\left\{ \begin{array}{cc} g_{T}=0\;and\;\frac{\partial g_{T}}{\partial t}=0 & \;\;\;\;if\;\tau \leq \mu |p|+\tau_{c}\;\text{(stick)}\\ \tau =\left(\mu |p|+\tau_{c}\right)\frac{\frac{\partial g_{T}}{\partial t}}{|\frac{\partial g_{T}}{\partial t}|} & if\;\tau >\mu |p|+\tau_{c}\left(slip\right) \end{array}\right.,$$

where μ is the coefficient of friction and $\tau_{c}$ is the fault cohesion. Physically, equation (4.8) states that if the contact traction does not exceed the counteracting frictional and cohesive stresses, then the fault is not allowed to tangentially slip any further after initial contact ( $g_{T}$ = 0). Meanwhile, if the contact traction does exceed both the opposing cohesive and frictional stresses, then the fault is allowed to slip with the magnitude and direction described by Amontons–Coulomb constitutive law.

The problem is solved using a four-stage double-loop gap-based augmented Lagrangian algorithm to fully resolve the traction and aperture distribution of the fault. In this method, gaps are augmented rather than contact tractions, and the normal and tangential Lagrange multipliers are defined, respectively, as


(4.9) $$\lambda_{N}=\epsilon g_{N}^{*}\;\;\;\;\text{and}\;\;\;\;\lambda_{T}=\epsilon g_{T}^{*},$$

where $g_{N}^{*}$ and $g_{T}^{*}$ are the augmented normal and tangential gaps, respectively. The algorithm is described in detail by Nejati [53,63].

Displacement of the fault is computed numerically, using the finite element method, by solving the PDEs in §4*a*–*c*. The work performs a quasi-static analysis of the fault slip, considering static friction, whereby fluid pressure is transient and the displacement is evaluated quasi-statically at each fluid pressure time step. A time step of 0.657 $\times \;10^{6}$ s was identified from a study of fault slip convergence for different time steps and used in this work.

### (d) Moment release and moment magnitude

The scalar seismic moment, $M_{0}$ , is given by [64]


(4.10) $$M_{0}=G\times A\times d,$$

where G is the shear or rigidity modulus, A is the area of the fault in which the rupture has occurred and d is the average slip across the fault surface. G is computed according to


(4.11) $$G=\frac{E}{2\left(1\;+\;\nu \right)},$$

where E is the Young’s modulus and ν is the Poisson’s ratio. The moment magnitude, $M_{w}$ , of an event can then be described by the relation [65]:


(4.12) $$M_{w}=\frac{2}{3}\log_{10}M_{0}-10.7.$$

## 5. Experimental setup

**Figure 1.**
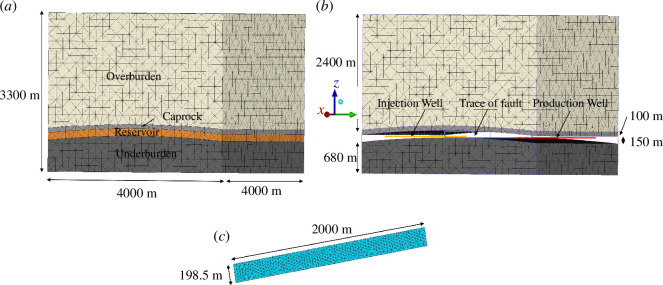
(*a*) A solid and wireframe view of the model showing the four rock layers (overburden is cream, caprock is light grey, reservoir is orange and underburden is dark grey). (*b*) A perspective front-right solid and wireframe view of the fault (cyan) contained with the reservoir unit and the injection well on the left of the domain (orange) and the withdrawal well on the right of the domain (red). The reservoir unit is removed in order to observe these. (*c*) A top view of the rectangular fracture surface.

A numerical model is constructed to study the injection, storage and withdrawal of hydrogen. The model has x × y × z dimensions of 4000 × 4000 × 3300 m and is shown in figure 1. The model contains an anti-clinal structure consisting of a caprock (light grey layer) and a reservoir unit (orange layer), bound from above by a low-permeability overburden (cream layer) and from below by a low-permeability underburden (dark grey layer). The mechanical, geological and geometrical properties of each of these layers, as well as fluid and fault properties, are given in table 3. The *in situ* stresses are applied symmetrically such that the *in situ* horizontal stresses at the mid-point of the reservoir unit are approximately 45 MPa, which obey the normal faulting regime that predominates in the central and southern North Sea [66,67]. Prescribed mechanical, geological and geometrical properties correspond to a generic North Sea reservoir where data were available (primarily an average from the Leman, Britannia and Goldeneye Fields; table 4). The fault properties are generalized.

**Table 3.** The mechanical, geometrical, geological, fluid dynamic and fault properties and the *in situ* stresses of the four different rock layers within the models.

property	overburden	caprock	reservoir	underburden
**mechanical**				
lithology	chalk	shale	sandstone	shale/coal
k (m^2^)	$3.0\times 10^{-15}$	$1.0\times 10^{-21}$	$1.0\times 10^{-13}$	$1.0\times 10^{-21}$
*φ*	0.30	0.05	0.15	0.05
h (m)	2300–2400	100	150	650–750
E (GPa)	$4.5\times 10^{9}$	$1.5\times 10^{10}$	$1.5\times 10^{10}$	$1.5\times 10^{10}$
ν	0.35	0.35	0.15	0.35
α	0.87	0.93	0.35	0.93
$\rho_{b}$ (kg/m^3^)	2080	2500	2500	2500
K (Pa)	$9.75\times 10^{9}$	$1.333\times 10^{10}$	$2\times 10^{10}$	$1.333\times 10^{10}$
$\sigma^{*}$ (Pa)	$1.0\times 10^{6}$	$1.3\times 10^{6}$	$2.0\times 10^{6}$	$1.3\times 10^{6}$
**fluid**				
fluid	brine	brine	hydrogen	brine
$\rho_{f}$ (kg/m^3^)	1058	1042	9	1037
μ ( μ Pa s)	561.0	357.5	10.4	296.0
c (Pa^−1^)	$4.47\times 10^{-10}$	$4.57\times 10^{-10}$	$6.67\times 10^{-8}$	$4.68\times 10^{-10}$
**stresses**				
$\sigma_{h}$ (MPa)	—	—	58.5	—
$\sigma_{H}$ (MPa)	—	—	58.5	—
$\sigma_{V}$ (MPa)	—	—	0.9800517	—
**fault**				
*l* (m)	—	—	2000	—
w (m)	—	—	183.8	—
$a_{f,r}$ (m)	—	—	$1.0\times 10^{-6}$	—
θ (°)	—	—	45	—
ψ (°)	—	—	0	—
x (m)	—	—	(0, 0, 2475)	—

The symbology in the table is as follows: k = permeability, ϕ = porosity, h = Thickness, E = Young’s modulus, ν = Poisson’s ratio, α = Biot coefficient, $\rho_{b}$ = rock Density, K = bulk modulus, $\sigma^{*}$ = uniaxial tensile strength, $\rho_{f}$ = fluid density, μ = fluid viscosity, c = fluid compressibility, $\sigma_{h}$ = minimum horizontal *in situ* stress at base, $\sigma_{H}$ = maximum horizontal *in situ* stress at base, $\sigma_{V}$ = vertical *in situ* stress at top, L = fault length, w = fault width, $a_{f,r}$ = residual aperture, θ = fault dip, ψ = fault orientation to North and x = fault centroid location (*x*, *y*, *z*). The properties herein are derived from the ‘overall range’ column of table 4.

**Table 4.** Literature review of values of reservoir, underburden/caprock and overburden properties and their sources or the sources from which data were taken to compute a value for that field.

property	Leman field	Britannia field	Goldeneye field	overall range
**reservoir**				
$E_{res}$ (GPa)	R: 0.32–12.68^a^ A: 7.18^a^	R: 20.6–86.2	R: 8.5–18.5	0.32–86.2
$K_{res}$ (mD)	A-s: 1.02–2.45 B-s: 6.03–15.60 C-s: 0.55–4.67	R: 0.1–200 A: 60 (E), 30 (W)	R: 700–1500 A: 790	0.1–1500
$\rho_{b,res}$ (kg/m^3^)	A-s: 2330–2600 B-s: 2280–2530 C-s: 2390–2620	R: 2350–2830 A: 2350 (T), 2650 (B)	R: 2013–2256	2013–2830
$\alpha_{res}$	—	—	—	—
$\nu_{res}$	R: −0.13 to 0.06^a^	R: 0.20–0.30^a^	R: 0.14–0.20	−0.13 to 0.30
$c_{r,res}$ (Pa^−1^)	A: 4.5 × 10^−10a^	R: 1.3 × 10^−11^–1.7 × 10^−10^ A: 3.9 × 10^−11^ (T), 3.4 × 10^−11^ (B)	A: 7.83 × 10^–11^	3.4 × 10^−11^−4.5 × 10^−10^
φ*_res_* (%)	A-s: 12.05–13.70 B-s: 13.54–15.06 C-s: 9.30–12.61	R: 10–18 A: 15	A: 25	9.3–25
**underburden/caprock**				
$E_{und}$ (GPa)	R: 1.45–3.37 (CM)^a^	R: 18–43 (KC)^a^	R: 5–15 (KC)^a^	1.45–43
$K_{und}$ (mD)	R: 0.0004–0.005 (CM)^a^ R: 0.09–6.62 (CM)^a^	R: 2 × 10^−6^ to 5 × 10^−6^ (KC)^a^	R: 7 × 10^−9^ to 5 × 10^−6^ (KC)^a^	7 × 10^−9^–6.62
$\rho_{b,und}$ (kg/m^3^)	R: 2430–2560 (CM)^a^	A: 2690 (KC)^a^	R: 1750–2150 (KC)^a^	1750–2690
$\alpha_{und}$	—	—	—	—
$\nu_{und}$	R: 0.14–0.37 (CM)^a^	R: 0.11–0.275 (KC)^a^	—	0.11–0.37
$c_{r,und}$ (Pa^−1^)	—	—	—	—
φ_*und*_ (%)	R: 0.51–4.14 (CM)^a^	R: 0–3.3 (KC)^a^ A: 2.5 (KC)^a^	R: 0.3–19.9 (KC)^a^	0–19.9
**overburden**				
$E_{over}$ (GPa)	—	R: 1–10^a^	R: 1–7 (NSC)^a^ A: 4–5 (NSC)^a^	1–10
$K_{over}$ (mD)	—	R: 1–5 (NSC)^a^	—	1–5
$\rho_{b,over}$ (kg/m ${\;}^{3}$ )	—	A: 2050 (T) (NSC)^a^ A: 2110 (B) (NSC)^a^	—	2050–2110
$\alpha_{over}$	—	R: 0.7–1.0 (NSC)^a^	R: 0.75–0.90 (NSC)^a^	0.7–1.0
$\nu_{over}$	—	R: 0.29–0.37 (NSC)^a^	—	0.29–0.37
$c_{r,over}$ (Pa^−1^)	—	—	—	—
φ_*over*_ (%)	—	R: 10–48 (NSC)^a^	R: 4–41 (NSC)^a^	4–48

The range for each property is given in the ‘overall range’ column, from which the values in table 3 were chosen.

The subscripts R, A, E, W, T, B, A-s, B-s, C-s denote range, average, east, west, top, bottom, A-sand, B-sand and C-sand, respectively.

KC, CM and NSC refer to Kimmeridge Clay, Coal Measures Rocks and North Sea Chalk, respectively. KC is the underburden rock for the Britannia and Goldeneye Fields, whereas CM is the underburden for the Leman Field. North Sea Chalk is the overburden rock for the Britannia and Goldeneye Fields.

The properties used in table 3 are derived from the ‘Overall Range’ column herein.

References from which each property was extracted were: $E_{res}$ —Monsees *et al*. [68], Camm *et al*. [69], Shell [70]; $K_{res}$ —Hillier [71], Camm *et al*. [69], Shell [72]; $\rho_{b,res}$ —Hillier [71], Camm *et al*. [69], Shell [73]; $\nu_{res}$ —Ji *et al*. [74], Dvorkin and Nur [75], Shell [70]; $c_{r,res}$ —Noy *et al*. [76], Camm *et al*. [69], Shell [70]; $\phi_{res}$ —Hillier [71], Camm *et al*. [69], Shell [72]; $E_{und}$ —Khandelwal and Singh [77], Sayers [78], Kumar *et al*. [79]; $K_{und}$ —Özgen Karacan [80], Huang *et al*. [81], Gaus *et al*. [82], Hawthorn [83]; $\rho_{b,und}$ —Khandelwal and Singh [77], Bell *et al*. [84], Hornby [85]; $\rho_{b,und}$ —Khandelwal and Singh [77], Sayers [78]; $\phi_{und}$ —Curtis *et al*. [86], Hawthorn [83], Huang *et al*. [81], Hornby [85]. The sources for the property values of the overburden and the caprock, assuming a chalk overburden and shale caprock as in the Britannia and Goldeneye fields are as follows: $E_{over}$ —Descamps *et al*. [87], Olsen [88]; $K_{over}$ —Keszthelyi *et al*. [89]; $\rho_{b,over}$ —Japsen [90]; $\nu_{over}$ —Olsen *et al*. [91], Gommesen *et al*. [92]; $\nu_{over}$ —Gommesen *et al*. [92]; $c_{r,over}$ —Alam *et al*. [93]; $\phi_{over}$ —Keszthelyi *et al*. [89], Gommesen *et al*. [92]; $\alpha_{cap}$ —Thompson *et al*. [94]. The remaining caprock properties: $E_{cap},K_{cap},\rho_{b,cap},\nu_{cap},c_{r,cap}$ and $\phi_{cap}$ are set to be equal to the underburden properties.

^a^ Denotes that the value is not specifically for that field but is the value for the equivalent lithological unit in another geographical location or outcrop or if that was not available, the value of a similar rock type.

Gaseous hydrogen is injected into the sandstone reservoir unit through a single horizontal wellbore that penetrates the whole length of the model in the y -direction, at a depth of 2475 m, 500 m away in the x -direction from the left boundary of the model (figure 1). Simulations are run for a total time of 3 years. In each annual cycle, the hydrogen is injected for a total time of five months at a constant injection rate of 20 m^3^/s. After the five-month injection period, the hydrogen remains in place with no withdrawal or storage taking place for a total of two months (5 256 000 s). Finally, the hydrogen is withdrawn for the remaining five months of the year via a second horizontal wellbore, also at a depth of 2475 m and 500 m away from the right boundary of the model in the negative x -direction. Therefore, the injection and withdrawal wells are separated by a distance of 3000 m in the x -direction. The withdrawal rate is set to a constant 17 m^3^/s, such that the withdrawal rate is 85% of the injection rate, accounting for the fact that not all the injected hydrogen will be recoverable. The chosen injection rate may at first seem large because the density of the injected hydrogen is extremely low relative to other geo-energy fluids. However, in order to store sufficiently large masses of hydrogen to comprise a significant and useful proportion of the energy mix, in short time periods of months, the injection rate will coincidentally have to be large.

The simulation is composed of the following two stages: (i) a pre-injection equilibration period that allows the model to reach the initial pressures set, and (ii) the injection period, lasting five months, a storage period lasting two months and a withdrawal period lasting five months. During the second stage, the fault slip is monitored.

The model contains a single fault (figure 1), modelled as a rectangular flaw of dimensions 2000 × 198.5 m, and centred at a depth of 2475 m, such that the fault resides in the reservoir unit. The fault orientation in the final simulations (§6) is described by a strike of 45°, a dip of 45° and a dip direction of 135°, whereas the fault orientation in the mesh convergence simulations (§5c) is described by a strike of 0°, a dip of 45° and a dip direction of 90°. The fault terminates at the top 10 m below the caprock–reservoir interface and at the bottom 10 m above the reservoir–underburden interface. The fault in the final simulations (§6) is defined by the following four points: (−760, 654, −2410), (−654, 760, −2540.00), (654, −760, −2410) and (760, −654, −2540), whereas in the mesh convergence simulations (§5c), it is defined by the following points: (−1000, −75, −2410), (−1000, 75, −2540), (1000, −75.00, −2410) and (1000, 75, −2540.00).

### (a) Mesh discretization

The rock mass is spatially discretized with isoparametric linear tetrahedra internally, and with isoparametric linear triangles on the model boundaries (figure 1) and fault. The model contains 191 513 tetrahedral volume elements and 14 509 triangular surface elements, including 651 on each fault face.

**Figure 2.**
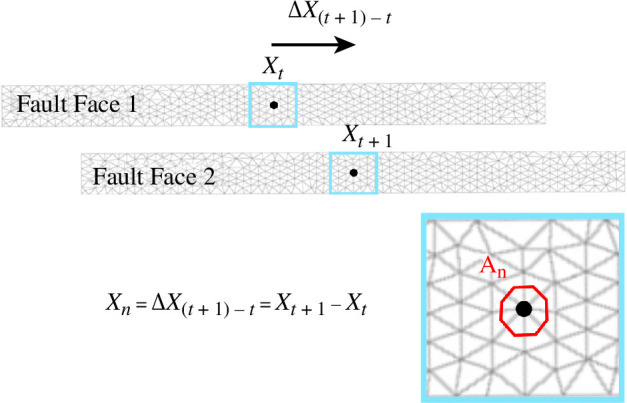
Pictoral representation of equation (5.1). The displacement of a node, n , from its initial position at a time step, t , is denoted by $x_{t}$ , and the displacement of a node, *n*, from its initial position at a time step, t+1 , is denoted by $x_{t+1}$ and thus $x_{t+1}$ − $x_{t}$ represents the node slip between the two time steps, $x_{n}$ . The area, $A_{n}$ , represents the area enclosed by the red boundary.

The faults are modelled as two rectangular surfaces, initially coincident and connected at the fault tips, making a void in the mesh. At each time step, the slip is computed at each of the nodes describing the fault surface and is subsequently averaged across all tip nodes using a weighted average dependent on the surrounding node area, which is the area contained within the zone bounded by the three mid-side nodes of the three lines connecting to that node of interest (figure 2), according to


(5.1) $$\sum_{n=1}^{N}x_{n}\cdot \frac{A_{n}}{A_{T}},$$

where N is the total number of nodes on the fault surface, $x_{n}$ is the slip at the node of interest, $A_{n}$ is the area surrounding the node of interest and $A_{T}$ is the total fault surface area. The distance between the fault surfaces, including aperture, shear and dilation, varies from node to node during the simulation and is an emerging property of the system.

### (b) Fault slip comparison

**Figure 3.**
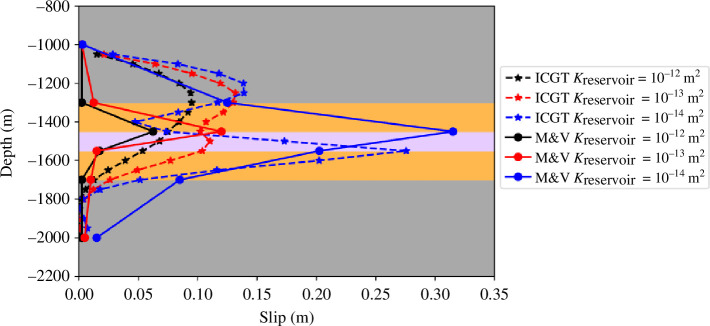
Model depth versus slip along the fault for different reservoir permeabilities. The results are presented after injection times of approximately 110, 280 and 1400 days in the 10^−12^, 10^−13^ and 10^−14^ m^2^ cases, respectively, as in the numerical comparison paper. The fault exists with vertical coordinates between 1000 and 2000 m. The solid black line represents the numerical solution of Mortezaei & Vahedifard [37], and *e* denotes elements. The dashed lines represent results generated from the ICGT.

Burtonshaw *et al*. [95] conducted a comparison study, in which the predictions of the ICGT were compared against the predictions of COMSOL Multiphysics for the computation of fault slip during a case of CO_2_ injection from the literature [37]. Mortezaei & Vahedifard [37] constructed a two-dimensional FEM 2000 × 2000 m thermo-hydro-mechanical model of a layer cake subsurface system consisting of the following five layers: an upper aquifer, an upper caprock, a reservoir, a lower caprock and a lower aquifer unit. The model contains one fault of length 1000 m and dip 80°, which penetrates to some extent all five structural units. CO_2_ was injected at a rate of 0.02 kg/s. They performed a parametric study, varying the permeability and porosity of the reservoir units and subsequently assessing the change in the slip along the fracture. In the work of Burtonshaw *et al*. [95], the model was replicated in the ICGT and extended to three dimensions, and a hydromechanical study was conducted. The thermal aspect of the work in Mortezaei & Vahedifard [37] was partially accounted for by assigning fluids of different densities, viscosities and compressibilities to each lithological unit depending on the corresponding temperature and pressure at the mid-depth of each unit. Burtonshaw *et al*. [95] found that the discrepancy between the peak slip across the fault surface between the ICGT and COMSOL results (figure 3) was small and within the centimetre scale (3, 1 and 5 cm in the 10^−12^, 10^−13^ and 10^−14^ m^2^). Differences in slip elsewhere along the fault surface can be attributed to differences in the modelling procedures: (i) two-dimensional (COMSOL) versus three-dimensional (ICGT), (ii) thermo-hydro-mechanical (COMSOL) versus hydromechanical (ICGT), (iii) potential differences in the methodology for slip computation, and (iv) constant apertures (COMSOL) versus geomechanically controlled apertures (ICGT). A host of further published validations, comparisons and studies for the ICGT in relevant fields applicable to the work herein, such as carbon dioxide sequestration and fracture physics have been performed [59].

### (c) Mesh effects

#### (i) Domain mesh effect

**Figure 4.**
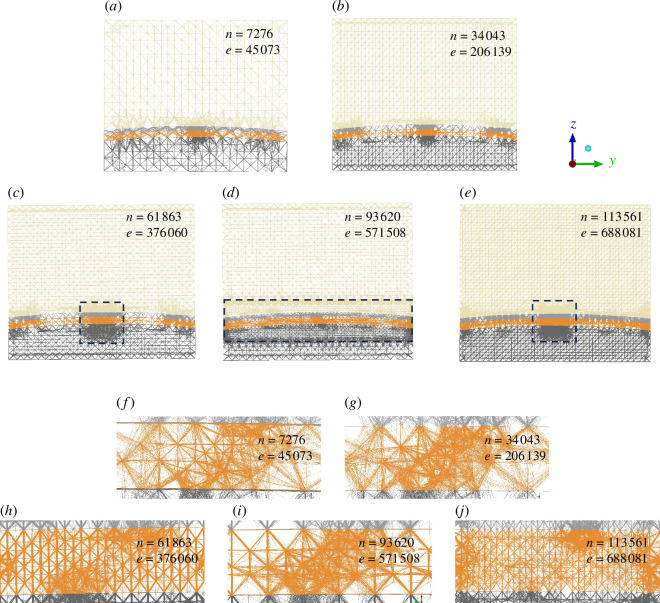
(*a*–*e*) Wireframe views of the five versions of the model for each refinement case in table 5. The dashed boxes denote the zones of increased refinement. (*f–j*) Zoomed views of the mesh at the centre of the reservoir unit.

A total of five separate simulations (table 5) in which the refinement of the bulk rock mesh is varied between each simulation were performed. These simulations used meshes with element numbers between approximately 45 000 and 690 000 and node numbers between approximately 7300 and 110 000. Figure 4 shows the refinement for each of the cases. In the cases with 7276 and 34 043 nodes, the entire domain is refined equally. In the cases with 61 863 and 113 561 nodes, the domain is additionally refined in a box of dimensions 2500 × 400 × 450 m centred at the fault centroid such that the fault is fully encompassed by a region of high refinement. In the case with 93 620 nodes, the domain is refined additionally in a larger box of dimensions 4000 × 4000 × 650 m, also centred at the fault centroid.

**Table 5.** Five simulation cases run to examine the effect of domain refinement on the slip solution.

simulation case	nodes	elements	fracture elements
case 1	7276	45 073	701
case 2	34 043	206 139	668
case 3	61 863	376 060	732
case 4	93 620	571 508	682
case 5	113 561	688 081	1202

**Figure 5.**
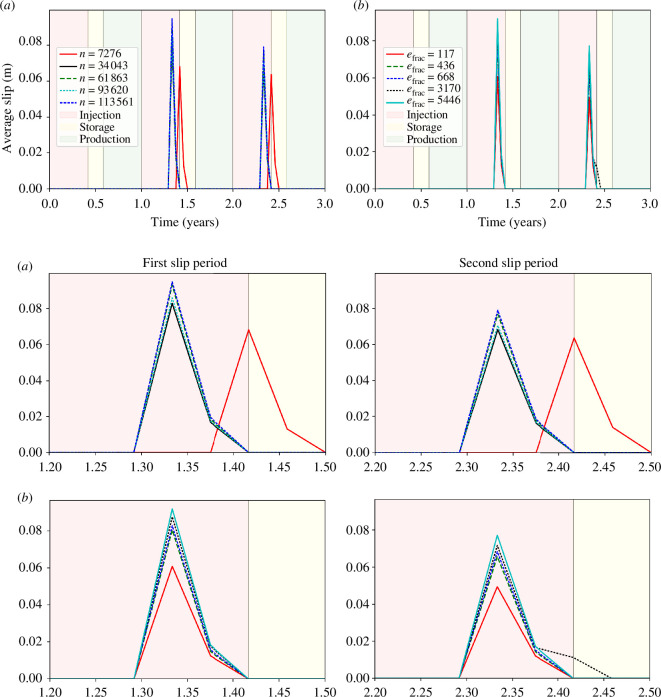
Plots of average slip as a function of operation time for (*a*) five cases of different bulk rock/domain refinements represented by different numbers of nodes and (*b*) five cases of different fault refinements represented by different numbers of fault elements showing two distinct slip events. A zoomed view of the two slip periods for both (*a*) and (*b*) are shown.

Plots of average slip as a function of time since the onset of the first injection period for each refinement case are shown in figure 5*a*. The solution converges to within 1 cm provided that the number of elements and the number of nodes are at least 200 000 and 34 000, respectively. Therefore, the remaining simulations in this work will use the mesh with 206 139 elements and 34 043 nodes. Finer refinement than this increases the simulation time to nearly a week for less than a centimetre accuracy improvement in solution. Predicting fault slip with sub-centimetre scale accuracy with any numerical model is also not feasible, and thus it is justified to use case 3. The case with only 7276 nodes is offset because the injection pressure, in this case, has not converged.

#### (ii) Fault mesh effect

**Figure 6.**
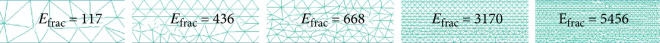
Mesh of the fault surface for the five versions of the fault in the model for each refinement case in table 6, for varying amounts of fracture elements *E*_frac_.

Five simulations (table 6) in which the refinement of the fault mesh is varied are performed. In each case, the entire domain is refined using the same refinement as case 2 from §5c(i). These simulations used fault meshes with element numbers between approximately 110 and 5500 elements. Figure 6 shows the refinement for each of the cases.

**Table 6.** Five simulation cases run to examine the effect of fracture refinement on the slip solution.

simulation case	nodes	elements	fracture elements
case 1	16 594	100 800	117
case 2	17 960	109 517	436
case 3	34 043	206 139	668
case 4	41 842	251 167	3170
case 5	91 641	546 919	5456

Plots of average slip as a function of time since the onset of the first injection period for each fault refinement case are shown in figure 5*b*. The solution again converges to within 1 cm provided that the number of elements is at least approximately 440. Only in the case of a fault with only 117 elements did the slip solution significantly diverge, predicting 3 cm less slip during the first and second slip events. Therefore, the remaining simulations use a ‘refined’ fault mesh with 668 elements. Finer refinement than this, such as in the case with 5456 fault elements, leads to a substantially longer simulation time, but leads to a 1 cm accuracy improvement in the slip solution.

### (d) Numerical experiments

This section outlines the various numerical simulations that were performed in this work. Four sets of simulations are performed in which four simulations are run per set. Each set corresponds to a particular mechanical property, either Young’s modulus, Poisson’s ratio, Biot coefficient or permeability. Within each set, each one of these properties in the reservoir unit is parametrically varied four times in four separate simulations so that the influence of each mechanical property on induced fault slip can be studied. Table 7 summarizes the test cases for the simulations. The values in each case are selected to cover the full range of potential values that each property was found to take in various depleted North Sea oil and gas fields.

**Table 7.** Mechanical properties of the different simulation cases.

simulation	default case	case 2	case 3	case 4
$E_{res}$ (GPa)	15	10	40	60
$k_{res}$ (mD)	100	5	25	800
$\alpha_{res}$	0.35	0.25	0.65	0.90
$\nu_{res}$	0.15	0.00	0.10	0.30

*E* = Young’s modulus, *k* = permeability, *α* = Biot coefficient, *ν* = Poisson’s ratio, *c*_*r*_ = rock compressibility.

The subscript ‘res’ refers to the reservoir unit.

## 6. Results

### (a) Young’s modulus

**Figure 7.**
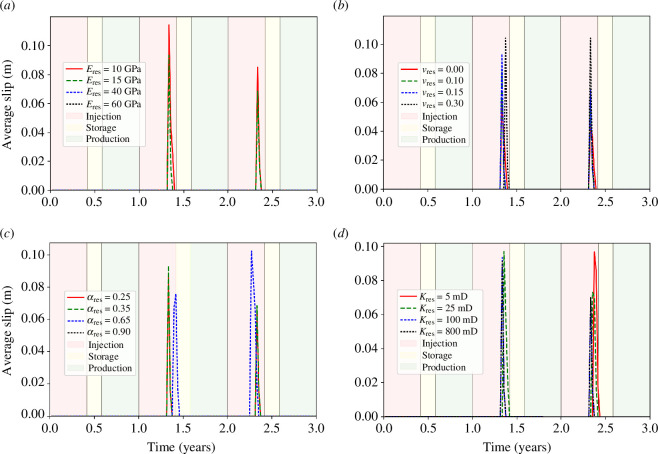
Average slip as a function of operation time for four cases of varying reservoir: (*a*) Young’s modulus, (*b*) Poisson’s ratio, (*c*) Biot coefficient and (*d*) permeability. Zoomed views of the slip peaks can be found in figure 8.

**Figure 8.**
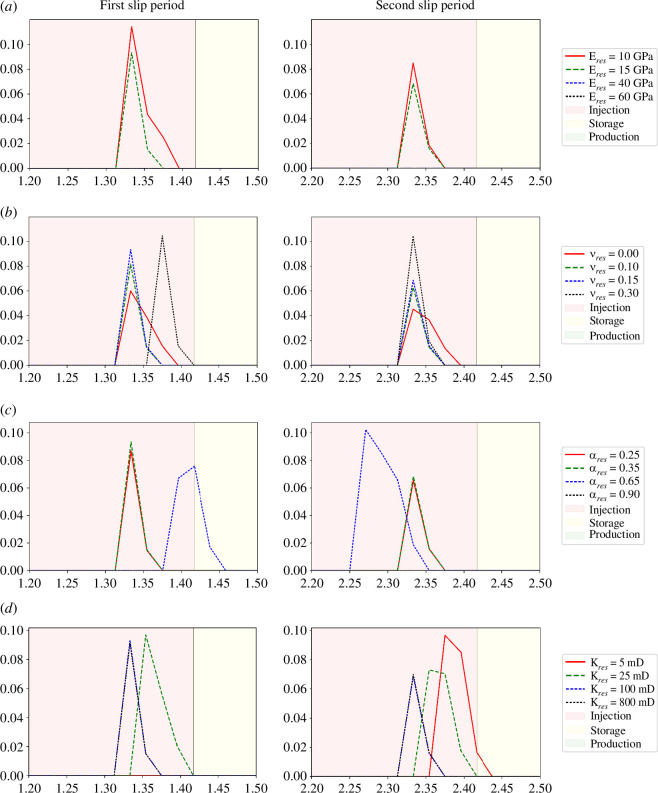
Zoomed views of the two distinct slip periods as a function of operation time from figure 7 for four cases of varying reservoir: (*a*) Young’s modulus, (*b*) Poisson’s ratio, (*c*) Biot coefficient and (*d*) permeability.

The Young’s modulus of the reservoir sandstone is varied between 10 and 60 GPa, and the influence on average slip as a function of time is shown in figures 7*a* and 8*a*. It is found that as reservoir Young’s modulus increases, the magnitude of the fault slip typically decreases. For the 10, 15, 40 and 60 GPa cases, the number of distinct slip events is 2, 2, 0 and 0, respectively. For the first slip event during the second injection period, the fault slip for the 10, 15, 40 and 60 GPa cases is 11.42, 9.34, 0 and 0 cm, respectively. For the second slip event during the third injection period, the fault slip for the 10, 15, 40 and 60 GPa cases is 8.50, 6.85, 0 and 0 cm, respectively. The Young’s modulus therefore has a significant effect on slip: for high reservoir stiffnesses, we can expect the fault to be completely stable, whereas for low reservoir stiffnesses, substantial motion could be expected with these injection and properties.

**Figure 9.**
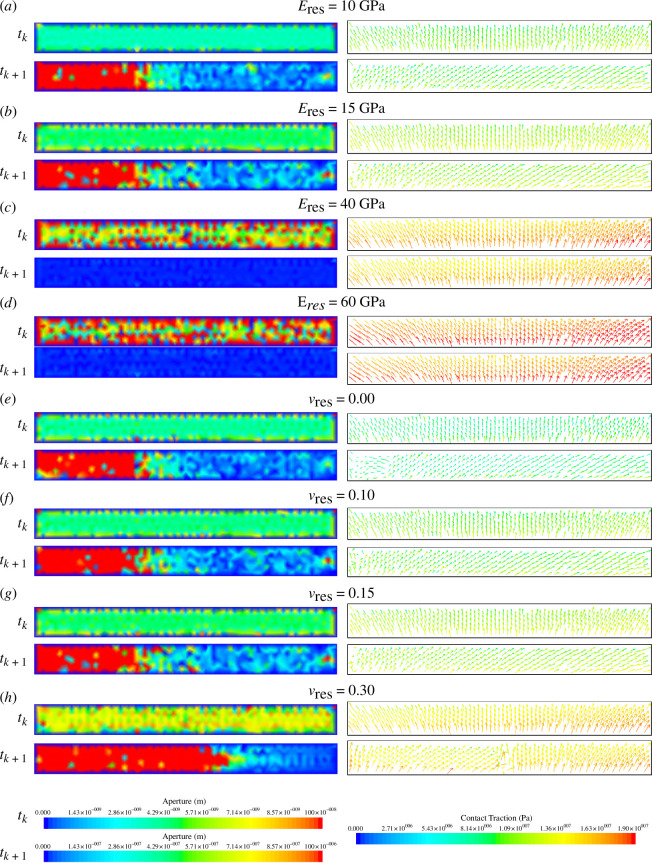
The fault aperture (left) and contact traction (right) one time step before ( $t_{k}$ ) and after ( $t_{k+1}$ ) the time of peak slip for the first slip event during the second injection period for four cases of varying reservoir (*a–d*) Young’s modulus and (*e–h*) Poisson’s ratio.

**Figure 10.**
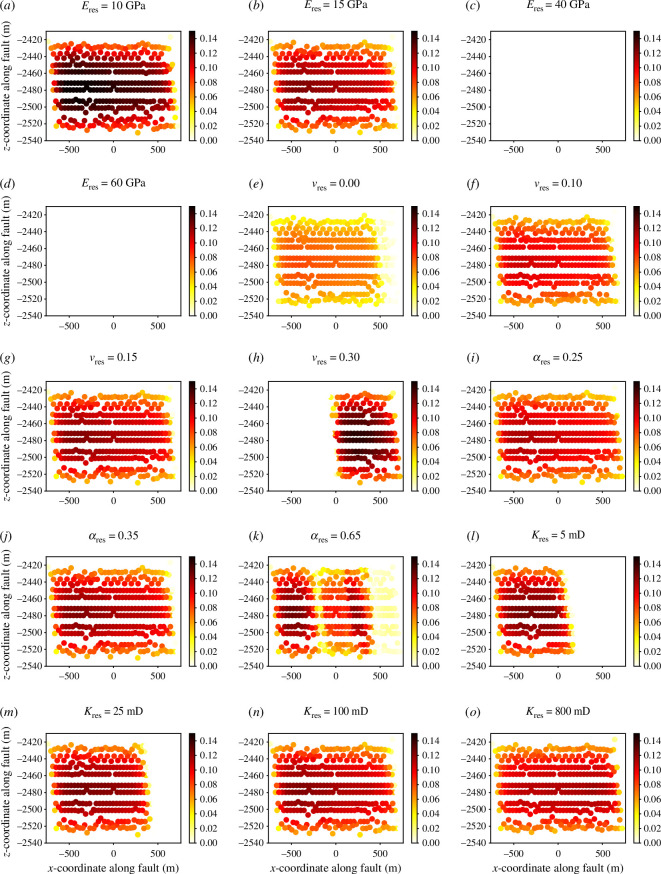
The tangential fault slip at each node at the time of peak slip for the first slip event during the second injection period for cases of varying reservoir: (*a–d*) Young’s modulus, (*e–h*) Poisson’s ratio, (*i–k*) Biot coefficient and (*l–o*) permeability.

The contact traction, fault aperture and fault slip at each node on the fault surface for the four different cases are shown in figures 9 and 10, respectively. Prior to the fault slipping, the contact tractions and fault aperture remained fairly uniform, but a sudden change in contact traction and a sudden increase in aperture were observed at the time of the events. Figure 9 shows that as Young’s modulus increases, the magnitude of the contact traction increases. Figure 9 shows that prior to the event, as Young’s modulus increases, the fault aperture increases, with the fault becoming progressively more open. However, it is also noted that as Young’s modulus increases, the change in the average fault aperture between the time step before and at the slip event decreases, and the aperture after the event decreases for increasing Young’s modulus. The larger increases in fault aperture reflect a larger reduction in normal contact tractions. As the contact between the two fault surfaces is reduced, the greater the propensity there is for the fault to slip tangentially (figure 10), as there is less friction between the two fault surfaces. In the cases with a reservoir Young’s modulus of 40 and 60 GPa, there is no change in the fault aperture (figure 9). This is reflected in the fact that no change in the contact traction is observed (figure 9). The contact traction is large and supplied by all normal traction, which keeps the fault closed and locked; thus, no change in tangential traction is observed.

**Figure 11.**
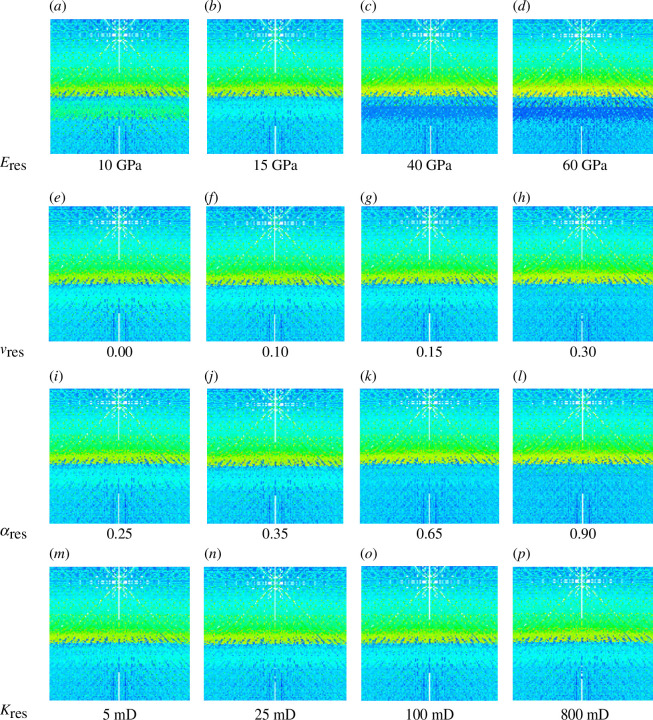
The effective strain over a vertical interval containing the anticlinal caprock and reservoir units at the time of peak slip for the first slip event during the second injection period for cases of varying reservoir: (*a–d*) Young’s modulus, (*e–h*) Poisson’s ratio, (*i–l*) Biot coefficient and (*m–p*) permeability.

Furthermore, examining the stress–strain state at the time of slip, the strain induced in the reservoir unit increases significantly as Young’s modulus decreases (figure 11). For the 40 and 60 GPa cases, no strain is induced in the reservoir, and the lack of elastic deformation means that the fault geometry is not influenced, thus it is no surprise we observe no slip. Increased effective strain correlates with increased fault slip.

Taking into account equations (4.10) and (4.12) and assuming that all the slip is released instantaneously, the upper bound for the moment magnitude of the potential induced seismicity for the first slip event in the 10 and 15 GPa cases is 3.50 and 3.56, respectively. Therefore, despite the fault slip increasing as Young’s modulus decreases, the maximum potential seismicity decreases slightly as Young’s modulus decreases, below 40 GPa, above which no slip occurs. This is because the decrease in the rigidity modulus as Young’s modulus decreases outweighs the small-to-moderate increase in slip as Young’s modulus decreases.

### (b) Poisson’s ratio

The Poisson’s ratio of the reservoir sandstone is varied between 0.00 and 0.30, and the influence on average slip as a function of time is shown in figures 7*b* and 8*b*. It is found that as reservoir Poisson’s ratio increases, fault slip increases. For the first slip event during the second injection period, the fault slip for the 0.00, 0.10, 0.15 and 0.30 cases is 6.00, 8.16, 9.34 and 11.38 cm, respectively. For the second slip event during the third injection period, the fault slip for the 0.00, 0.10, 0.15 and 0.30 cases is 4.53, 6.29, 6.85 and 10.43 cm, respectively. The Poisson’s ratio therefore has a significant effect on slip: for higher reservoir Poisson’s ratio, we can expect nearly double the fault slip in the 0.30 case than in the 0.00 case.

The contact traction along the fault, the fault aperture and the fault slip at each node on the fault surface for the four different cases are shown in figures 9 and 10, respectively. Figure 9 shows that as Poisson’s ratio increases, the magnitude of the contact traction also increases. Figure 9 shows that as the Poisson’s ratio increases, the fault aperture prior to the slip event increases slightly, with the fault becoming more open with higher Poisson’s ratio. As the Poisson’s ratio increases, especially beyond ν = 0.15, the fault aperture after the slip event increases. A larger portion of fracture surface area opens in the ν = 0.30 case, not just the portion of the fault closest to the injection wellbore. Again, the larger change in fault aperture mirrors the larger fault slip. The larger the sudden increase in fault aperture, the larger the sudden reduction in normal contact traction and the greater propensity the fault has to slip tangentially. Interestingly, although the magnitude of the fault slip is the largest in the 0.30 case, the fault area in which the slip occurs is also the smallest. For approximately half of the fault, there is no tangential fault slip (figure 10) because the contact traction at this point is fully supplied by normal contact traction with no tangential component (figure 9) and the fault remains effectively closed in this zone.

Taking into account equations (4.10) and (4.12) and assuming that all the slip is released instantaneously, the upper bound for the moment magnitude of the potential induced seismicity for the first slip event in the 0.00, 0.10, 0.15 and 0.30 cases, respectively, is as follows: 3.47, 3.53, 3.56 and 3.38. Therefore, unlike the trend in the fault slip, the maximum potential seismicity does not progressively increase as Poisson’s ratio increases. In fact, the magnitude of the maximum potential seismicity was the smallest in the highest Poisson’s ratio case ( ν = 0.30), owing to the combination of the reduced slip area (figure 10) and the reduced rigidity modulus of the fault, and was largely insensitive in the range 0.00–0.15.

**Figure 12.**
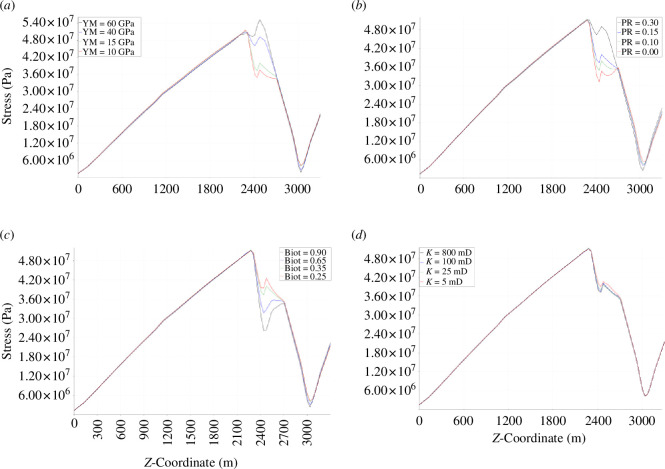
Plots of stress along the mid-depth of the reservoir unit in the *y*-direction for four cases of varying reservoir (*a*) Young’s modulus, (*b*) Poisson’s ratio, (*c*) Biot coefficient and (*d*) permeability.

Examining the stress–strain state at the time of slip, the stress induced in the reservoir unit decreases significantly between the cases (figures 11 and 12). The stress along a vertical line from the top of the model to the bottom (through the centre of the model) at the reservoir unit depth is approximately 33 MPa in the ν = 0.00 case, whereas it increases to 47 MPa in the ν = 0.30 case. The stress along the mid-depth of the reservoir unit, either side of the fault, is approximately 42.5 MPa in the ν = 0.00 case, whereas it increases to 49.2 MPa in the ν = 0.30 case. The much lower stresses acting on the fault plane in the ν = 0.00, ν = 0.10 and ν = 0.15 cases mean that the effective stresses are lower, and thus the frictional resistance required to prevent tangential fault slip is lowered according to the Coulomb criterion, making it easier for the fault to slip tangentially. Lower strain is also observed in the reservoir unit in the ν = 0.30 case, which again reinforces the observation of the lowest maximum potential magnitude of induced seismicity in this case. One would expect that for rare cases, where the Poisson’s ratio is above 0.30, the strains would be very low and the stresses very high, and the entire fault would be locked.

### (c) Biot coefficient

The Biot coefficient of the reservoir sandstone is varied between 0.25 and 0.90, and the influence on average slip as a function of time is shown in figures 7*c* and 8*c*. A systematic change in the slip with Biot coefficient is not found. For the first slip event during the second injection period, the fault slip for the 0.25, 0.35, 0.65 and 0.90 cases is 8.69, 9.34, 7.58 and 0 cm, respectively. For the second slip event during the third injection period, the fault slip for the 0.25, 0.35, 0.65 and 0.90 cases is 6.55, 6.85, 10.24 and 0 cm, respectively. For the first event, it can generally be observed that as Biot coefficient increases, the magnitude of the peak slip decreases: the slip is almost identical in the 0.25 and 0.35 cases, but decreases considerably by the 0.65 case, and then continues to decrease where the fault is completely stable by the 0.90 case. However, this trend is not repeated for the second event. At values of the Biot coefficient less than 0.65, the fault slip is not significantly dependent on the Biot coefficient. However, in the range of 0.65–0.90, the Biot coefficient significantly controls the fault slip.

**Figure 13.**
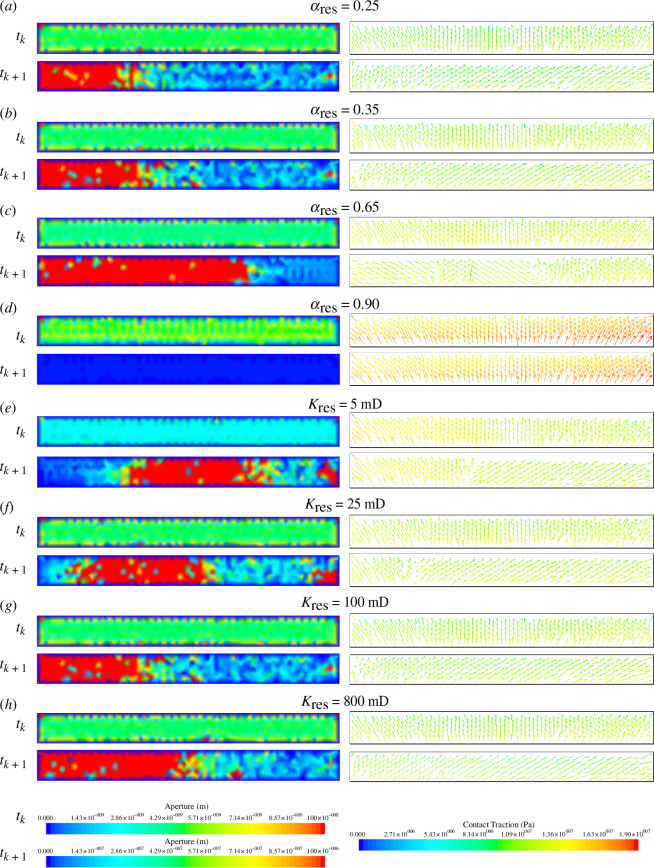
The fault aperture (left) and contact traction (right) one time step before ( $t_{k}$ ) and after ( $t_{k+1}$ ) the time of peak slip for the first slip event during the second injection period for four cases of varying reservoir: (*a–d*) Biot coefficient and (*e–h*) permeability.

The contact traction and the fault aperture, the fault slip at each node on the fault surface, the strain and the stress state for the four different cases are shown in figures 10–13, respectively. Figure 13 shows that as Biot coefficient increases, the magnitude of the contact traction also increases, although not significantly until the 0.90 case. Figure 13 shows that generally as the Biot coefficient increases, the fault aperture prior to the slip event increases, with the fault becoming progressively more open with higher Biot coefficient. As the Biot coefficient increases in the range of 0.25–0.65, the fault aperture after the slip event increases; however, above 0.65, the fault aperture remains closed. The slips at each individual node (figure 10) in the 0.65 case are more varied than those observed in the 0.25 and 0.35 cases, with the distribution of slip across the fault surface being less smooth. Furthermore, in the 0.65 case, it is observed that a region in which tangential slip is low, near the middle of the fault and near the right side (figure 10) has developed relative to the 0.25 and 0.35 cases. Therefore, it is likely that at values of the Biot coefficient between 0.65 and 0.90, the average fault slip will continue to decrease as these regions of the fault surface that are in stick grow in size, until a value of the Biot coefficient is reached, in which the entire fault surface area is in stick such as the 0.90 case.

Taking into account equations (4.10) and (4.12) and assuming that all the slip is released instantaneously, the upper bound for the moment magnitude of the potential induced seismicity for the first slip event in the 0.25, 0.35 and 0.65 cases, respectively, is as follows: 3.53, 3.56 and 3.42. Therefore, as with the fault slip, generally, the maximum potential seismicity decreases slightly as Biot coefficient increases. The strain in the reservoir unit is seen to be similar in the 0.25 and 0.35 cases, in which the slip is very similar, but the strain decreases as Biot coefficient increases above 0.35, reflecting the decreased fault slip with increasing Biot coefficient (figure 11).

### (d) Permeability

The permeability of the reservoir sandstone varies between 5 and 800 mD, and the influence on average slip as a function of time is shown in figures 7*d* and 8*d*. It is found that for the reservoir permeabilities tested, the magnitude of the induced fault slip is similar. For the first slip event during the second injection period, the fault slip for the 5, 25, 100 and 800 mD cases is 9.67, 9.70, 9.34 and 9.12 cm, respectively. A small trend is seen here where as permeability increases, the magnitude of the peak fault slip decreases. For the second slip event during the third injection period, the fault slip for the 25, 100 and 800 mD cases is 7.30, 6.85 and 6.99 cm, respectively. The second slip event for the 5 mD case did not occur by the end of the 3-year study period. The permeability therefore has no major effect on the magnitude of the slip, although it is generally seen that as the permeability increases, the magnitude of the average fault slip decreases slightly. The permeability does have an effect on the time in which the slip occurs. The higher the permeability of the reservoir unit, the earlier the induced slip occurs. This is because the fault pressure at a given time varies depending on the permeability. It takes longer for the fluid front from the injection wellbore to reach the fault and then leak-off into the fault when the permeability is smaller. The 800 and 100 mD cases coincide temporally, although one would expect the 800 mD case to have slipped earlier than the 100 mD case. The only reason this does not occur is because of the time step size, which is large enough to mask the small time difference. As such, the slip appears to be at identical times in the 100 and 800 mD cases, although if very small time steps were feasible, it would be initiated very slightly before (a few days). The slip event is only significantly delayed when the intrinsic rock permeability approaches the fracture permeability which is 1 mD. Therefore, when the intrinsic permeability is well in excess of the fracture permeability (the 800, 100 and 25 mD cases), the delay in the slip is minor, but when the intrinsic permeability is close to the fracture permeability (the 5 mD case), the slip event is significantly delayed. Therefore, the fracture permeability is likely to be a key parameter affecting the timing of the seismicity.

The contact traction along the fault, the fault aperture, the fault slip at each node on the fault surface, the strain and the stress state for the four different cases are shown in figures 10–12–13, respectively. The contact traction, aperture and strains show no significant differences in the 25, 100 and 800 mD cases, and in this case, the magnitude of the fault slip is not significantly impacted. The slip at each individual node across the fault surface (figure 10) shows an interesting trend, as the reservoir permeability decreases, the proportion of the fault in stick increases and the proportion of the fault in slip decreases. Taking into account equations (4.10) and (4.12) and assuming that all the slip is released instantaneously, the upper bound for the moment magnitude of the potential induced seismicity for the first slip event in the 5, 25, 100 and 800 mD cases is 3.42, 3.49, 3.56 and 3.55, respectively.

## 7. Discussion

As Young’s modulus increases, the average fault slip and maximum potential event magnitude decrease. For Young’s moduli over 40 GPa, the fault was completely stable. The strain induced in the reservoir unit decreased with increasing Young’s modulus.

The reduction in fault slip as Young’s modulus increased, found in this work, is in line with that found by Meng *et al*. [32] in their hydraulic fracturing work. They varied Young’s modulus of their one-unit formation between 5 and 50 GPa and studied the slip along a natural fracture that was spatially penetrated by their injection wellbore. The slip continuously decreased from a maximum of 12 cm in the 5 GPa case to 1–2 cm in the 50 GPa case, owing to a decrease in strain, which is what we observe in this hydrogen storage case (figure 11).

As Poisson’s ratio increases, the average fault slip increases. However, the maximum potential event magnitude was the smallest in the highest Poisson’s ratio case ( ν = 0.30) because a large proportion of the fault surface was found to be in stick. Therefore, the maximum potential event magnitude reduces with higher Poisson’s ratio.

The only other study examining the effect of Poisson’s ratio on fault slip during an injection procedure was that of Meng *et al*. [32] in their two-dimensional hydraulic fracturing work. They found that the fault slip is insensitive to changes in Poisson’s ratio in the range 0.1–0.3, whereas we found that in this range the average fault slip can vary between approximately 3–4 cm. The work here has the advantage of being three-dimensional, with a two-dimensional fault, so that the distribution of slip across the realistic two-dimensional fault surface can be studied. A two-dimensional model with a one-dimensional fracture surface is restricted to computing the fracture slip at just one point along the depth of the fault. Furthermore, the work is considerably different here in the sense that the pressure cycles up and down (injection and withdrawal), rather than systematically increasing, and that the fault does not, or comes close to, spatially intersect the injection wellbore, which is likely a different problem since the fault automatically becomes highly pressurized.

No systematic trend in fault slip with increasing Biot coefficient was found. The fault was completely stable in the highest Biot coefficient case ( α = 0.90). The magnitude of the average fault slip was nearly identical in the α = 0.25 and α = 0.35 cases and was not significantly different in the 0.65 case. Therefore, the fault slip only differed significantly for high Biot coefficient cases (above α = 0.65), where the fault becomes more stable.

The Biot coefficient of the reservoir formation is varied, and the influence on fault slip is assessed. Hui *et al*. [33] simulated three cases of Biot coefficient of 0.22, 0.44 and 0.66 and assessed the change in CFS along a pre-existing fault during hydraulic fracturing. They found that the CFS was effectively the same, with only 0.4 MPa difference between the 0.22 (2.7 MPa) and 0.66 (2.3 MPa) cases, implying that the fault is likely to have similar stability irrespective of the Biot coefficient within this range. The findings of this study are consistent with less than 2 cm difference in slip between the 0.25 and 0.65 cases for the first event. In fact, the slight decrease in CFS as Biot coefficient increases implies a slight decrease in expected fault slip as Biot coefficient increases, as observed for the first event in the present paper. However, given that the work of Hui *et al*. [33] involves large networks of hydraulic fractures, the proximity of this work to that is uncertain. Fan *et al*. [36] concluded the same as Hui *et al*. [33], but their work examined the change in CFS along a basement fault, which again is a different problem to the one studied herein.

The magnitude of the average fault slip was largely insensitive to changes in intrinsic permeability, although as permeability increased, the fault slip tended to decrease slightly. The time of the first event was dependent on intrinsic permeability, with the fault first slipping at later times with smaller permeabilities. The delay was stronger when the intrinsic permeability was closer to the fracture permeability, potentially indicating that the fracture permeability has a strong control over the timing of the seismicity.

Comparing these results to the literature, Mortezaei & Vahedifard [37] injected CO_2_ for sequestration with no withdrawal in a two-dimensional model. They found that as permeability decreased, the magnitude of the fault slip increased, which is what we observe here, although the relationship is not strong. They also found that the timing of the first rupture was delayed as permeability decreased, which is also observed herein. This work, relative to that, benefits from the fact that the slip is computed across a two-dimensional fault surface, showing a full spatial distribution of fault slip. The two-dimensional nature of the work in Mortezaei and Vahedifard [37] constrains the fault to a one-dimensional nature, meaning the slip is essentially being computed at just one point along the fault at each depth interval.

The maximum potential event magnitude found varied between no event and a 3.56 magnitude event. The large maximum event magnitude found can be attributed to a number of factors, including a large fault area found to be in slip and the large injection rates used. Unlike other geo-energy applications, with hydrogen storage, the injection rates used will need to be high. Hydrogen’s very low density at typical reservoir temperatures and pressures of less than 10 kg/m^3^ imply that large volumes of fluid must be delivered to the rock in order to store the masses necessary. It may be the case that distributing the injection volume through many different wellbores and through different well patterns may reduce these magnitudes, although this would be the subject of future work. The injection rates used herein represent a worst-case scenario. Notice that these results are based on the assumption that the peak slip is all released instantaneously and not uniformly across one computational time step (7 days). The results assume that there is a single fluid in the reservoir and that there is no plastic deformation of the caprock during the storage cycle.

## 8. Conclusions

In this work, a three-dimensional hydromechanical, linear elastic, fracture mechanics-based numerical simulator parametrically studies the effects of mechanical properties of the reservoir rock on fault slip and induced seismicity. The behaviour of a four-layer anti-clinal model with a single inclined through-going fault in the reservoir is investigated. Gaseous hydrogen is stored for three annual cycles, consisting of five-month injection, two-month storage and five-month withdrawal periods. Five parameters are investigated, namely Young’s modulus, Poisson’s ratio, Biot coefficient and intrinsic permeability of the reservoir unit, which were varied within a range consistent with the expected properties at North Sea fields. The effect of these properties is quantified in terms of slip, and the maximum potential seismic event magnitude is approximated. It was found that Young’s modulus and Poisson’s ratio had the strongest control on the fault slip, and Biot coefficient to a lesser extent. The magnitude of the fault slip was insensitive to changes in reservoir permeability. The reservoir rock with high Young’s modulus (>40 GPa), high Poisson’s ratio (>0.30) and high Biot coefficient (>0.65) was found to yield the minimum slip during the simulation of hydrogen storage.

## Data Availability

This article has no additional data.
